# Regional differences in characteristics, service use, and outcomes in patients receiving home visit care in Japan

**DOI:** 10.1093/geroni/igaf122.3648

**Published:** 2025-12-31

**Authors:** Jun Komiyama, Yu Sun, Masao Iwagami, Nobuo Sakata, Satoru Yoshie, Tomoko Ito, Kimikazu Kashiwagi, Nanako Tamiya

**Affiliations:** University of Tsukuba, Tsukuba, Ibaraki, Japan; University of Tsukuba, Tsukuba, Ibaraki, Ibaraki, Japan; University of Tsukuba, Tsukuba, Ibaraki, Japan; University of Tsukuba, Tsukuba, Ibaraki, Ibaraki, Japan; University of Tsukuba, Tsukuba, Ibaraki, Ibaraki, Japan; University of Tsukuba, Tsukuba, Ibaraki, Japan; National College of Nursing, Kiyose, Tokyo, Japan; University of Tsukuba, Tsukuba, Ibaraki, Japan

## Abstract

**Aim:**

Physician-led home-visit care has become essential in the super-aged society of Japan. To develop region-specific service systems, understanding regional differences in patient profiles is essential. This study aimed to describe regional differences in patient characteristics, service utilization, and outcomes, and to assess the associations between regional healthcare resources and patient outcomes.

**Methods:**

This retrospective study used linked data from the National Database of Health Insurance Claims and Specific Health Checkups and the Japanese Long-term Care Database (April 2020–March 2021). Patients aged ≥65 years receiving regular physician-led home visits in October 2020 were included. Analyses were conducted by prefecture and region. Associations between regional healthcare resources and patient outcomes were assessed using Spearman’s correlation coefficients (ρ).

**Results:**

Among 337,863 patients, regional variations were observed. Western Japan had higher prevalence of cancer, heart disease, respiratory disease, and orthopedic disorders, whereas eastern Japan showed higher rates of high care-need levels and home nursing utilization. Hospitalization risk was higher in western Japan. In-home deaths were more frequent in eastern Japan. Hospital bed density was positively correlated with hospitalization risk (ρ = 0.62) and negatively with 6-month mortality risk (ρ=-0.44). Utilization of enhanced home care support clinics/hospitals was moderately associated with higher in-home death risks (ρ = 0.49).

**Conclusions:**

Substantial regional differences exist in patient characteristics, service utilization, and outcomes among older adults receiving physician-led home-visit care. Healthcare resources were strongly associated with hospitalization and end-of-life care, highlighting the need for region-specific strategies to optimize healthcare delivery and resource allocation for the super-aged society of Japan.

